# Motor imagery affects both cortical and spinal circuitry: a transcranial and trans-spinal magnetic stimulation study

**DOI:** 10.3389/fncir.2026.1809125

**Published:** 2026-04-30

**Authors:** Asma Benachour, Nikolay Syrov, Mikhail Lebedev

**Affiliations:** 1 Vladimir Zelman Center for Neurobiology and Brain Rehabilitation, Skolkovo Institute of Science and Technology, Moscow, Russia; 2 Computational Neurology Group, Faculty of Medicine, Ruhr University Bochum, Bochum, Germany; 3 Research Center in the Field of Artificial Intelligence, Lomonosov Moscow State University, Moscow, Russia; 4 Faculty of Mechanics and Mathematics, Lomonosov Moscow State University, Moscow, Russia

**Keywords:** corticospinal tract, corticospinal excitability, motor imagery, non-invasive stimulation, spinal pathway, TMS, trans-spinal magnetic stimulation

## Abstract

**Introduction:**

Motor imagery (MI), the mental rehearsal of movement without physical execution, is a key technique in brain-computer interfaces (BCIs), known for eliciting cortical modulations similar to those exhibited during real movement. Beyond cortical effects, MI could also modulate spinal cord processing, which offers additional potential for neurorehabilitation in conditions like spinal cord injury (SCI) and stroke, where BCIs are used for therapy.

**Material and methods:**

To investigate the interactions of MI with both the cortex and the spinal cord, we employed both transcranial magnetic stimulation (TMS) and trans-spinal magnetic stimulation (TSMS) while recording brain and muscle activities.

**Results and conclusion:**

With proper coil orientation, TSMS elicited lateralized MEPs in ipsilateral forearm muscles at significantly shorter latencies than M1-evoked MEPs, confirming direct spinal cord activation. Importantly, right-hand kinesthetic MI selectively facilitated TSMS-evoked MEPs in the stimulated ipsilateral side only, providing direct evidence that MI modulates spinal cord excitability. Moreover, TSMS-evoked cortical responses were modulated by imagery, demonstrating that MI increases cortical processing of the ascending spinal volley. This within-group demonstration of MI affecting both cortical and spinal circuitry underscores its potential as a powerful strategy for BCI-driven neurorehabilitation, including pairing MI with spinal magnetic stimulation.

## Introduction

Motor Imagery (MI), the mental rehearsal of movement without execution (Lotze and Halsband, 2006), is a well-established paradigm in brain-computer interfaces (BCIs) that has been used for promoting neuroplasticity in motor rehabilitation (Liao et al., 2023; Teo and Chew, 2014). MI engages cortical networks largely overlapping with that of physical movement, including the primary motor cortex (Schnitzler et al., 1997), premotor areas (Decety, 1996), the supplementary motor area (Mehler et al., 2019), and posterior parietal cortices (Aflalo et al., 2015). Transcranial Magnetic Stimulation (TMS) has been effectively used for probing the cortex during MI; studies combining TMS with electroencephalography (EEG) showed the modulation of cortical activity during imagery and an increase in the amplitudes of motor evoked potentials (MEPs) in muscles associated with the imagined movements, suggesting the modulation of the corticospinal tract (Kasai et al., 1997; Facchini et al., 2002; Fadiga et al., 1998; Li et al., 2004).

While the cortical mechanisms underlying motor imagery (MI), reflected by an increased cortical excitability, are well established, less is known about how MI affects the spinal tract. Traditional techniques, such as H-reflex or F-wave, were used during imagery to investigate spinal modulation and led to various results (Bonnet et al., 1997; Hale et al., 2003; Nakagawa et al., 2018; Hara et al., 2010) as these methods indirectly probes the corticospinal pathway and are limited to monosynaptic reflex pathways, which are directed by presynaptic inhibition, leading to an unresolved question of whether MI truly reaches the spinal cord.

With the development of non-invasive modulation techniques, several approaches have also been used to probe and modulate the spinal cord, such as transcutaneous electrical spinal cord stimulation (tSCS), which has demonstrated the feasibility of noninvasively modulating spinal cord excitability and was later used effectively in rehabilitation alone or in combination with MI (Capozio et al., 2023; Moritz et al., 2024). Similarly, magnetic stimulation could also modulate spinal cord excitability. Trans- spinal magnetic stimulation (TSMS) is an approach that involves the delivery of brief magnetic pulses at the spinal cord level, and studies combining MI with magnetic spinal cord stimulation remain limited (Chalfouh et al., 2020; Knikou, 2014). In the BCI domain, a recent study demonstrated a non-invasive brain-spine interface (BSI) where EEG signals of MI of leg movements were decoded to control TSMS commands to the lumbar spine (Insausti-Delgado et al., 2022), eliciting muscle activation in the tibialis anterior muscle, hence demonstrating the feasibility of integrating MI with magnetic spinal stimulation. However, understanding the extent to which the spinal cord excitability is increased during imagery remains crucial for further clinical applications.

In the present study, we used TSMS and TMS to directly and independently probe the effects of MI at the spinal and cortical levels within the same participants, bypassing the limitations of classical indirect reflex measures. The use of a within-subject design allowed us to directly compare MEP latencies between spinal and cortical stimulation sites, providing a latency-based validation of the spinal origin of TSMS-evoked responses. Simultaneously, EEG recordings during TSMS allowed us to characterize ascending spinal volleys reaching the cortex (TSEPs) and their modulation by MI. Here, we hypothesize that [1] cervical TSMS delivered with a figure-of-eight coil would elicit lateralized motor-evoked potentials (MEPs) in ipsilateral upper-limb muscles, indicating focal spinal stimulation; [2] right-hand MI would selectively facilitate TSMS-evoked MEPs on the ipsilateral (right) side, but not on the contralateral (left) side; and [3] MI would modulate cortical responses to both TMS and TSMS, reflected in changes in the amplitudes of TMS-evoked potentials (TEPs) and TSMS-evoked potentials (TSEPs).

## Material and methods

Ethical approval was obtained from the Ethical Committee of the Skolkovo Institute of Science and Technology (No. 11 dated October 17, 2023), and the study was conducted following the principles of the Declaration of Helsinki. All participants provided written informed consent prior to their participation in this study.

### Experiment 1 (pilot study)

The pilot experiment aimed to investigate the feasibility of using the TMS figure-eight coil to stimulate the spinal cord and elicit lateralized MEPs in the ipsilateral arm and hand muscles. A total of 10 healthy participants (10 males, mean age 26.6; 19–34 years old, one left-handed) were recruited, all of whom met inclusion criteria of the absence of neurological, motor, psychiatric, and cognitive impairments. The exclusion and inclusion criteria conformed to the general TMS criteria (Rossi et al., 2021). Data from 2 participants were excluded due to issues related to data file acquisition.

#### TMS experimental design and setup

##### Spinal cord stimulation

The experimental design comprised four distinct stimulation conditions targeting two regions of interest: the right and left sides of the spinal cord at the C6-C7 vertebral level (corresponding to the C5 and C6 nerve roots). For each stimulation site, two experimental conditions were tested: resting and MI of closing and opening of the right hand.

Left-sided stimulation was used to assess both the lateral specificity of TSMS effects and the effector specificity of right-hand motor imagery at the spinal level.

Most participants were naïve to motor imagery tasks and had no prior experience with transspinal magnetic stimulation. Before the experimental session, all participants were familiarized with the imagery task. They first repeatedly opened and closed their right hand and were then instructed to imagine performing the same movement without any overt execution, focusing on the associated muscle- and tendon-related sensations. This procedure was used to encourage kinesthetic rather than visual motor imagery. Before proceeding, the experimenter verified that each participant was able to perform the task as instructed.

**Figure 1:**
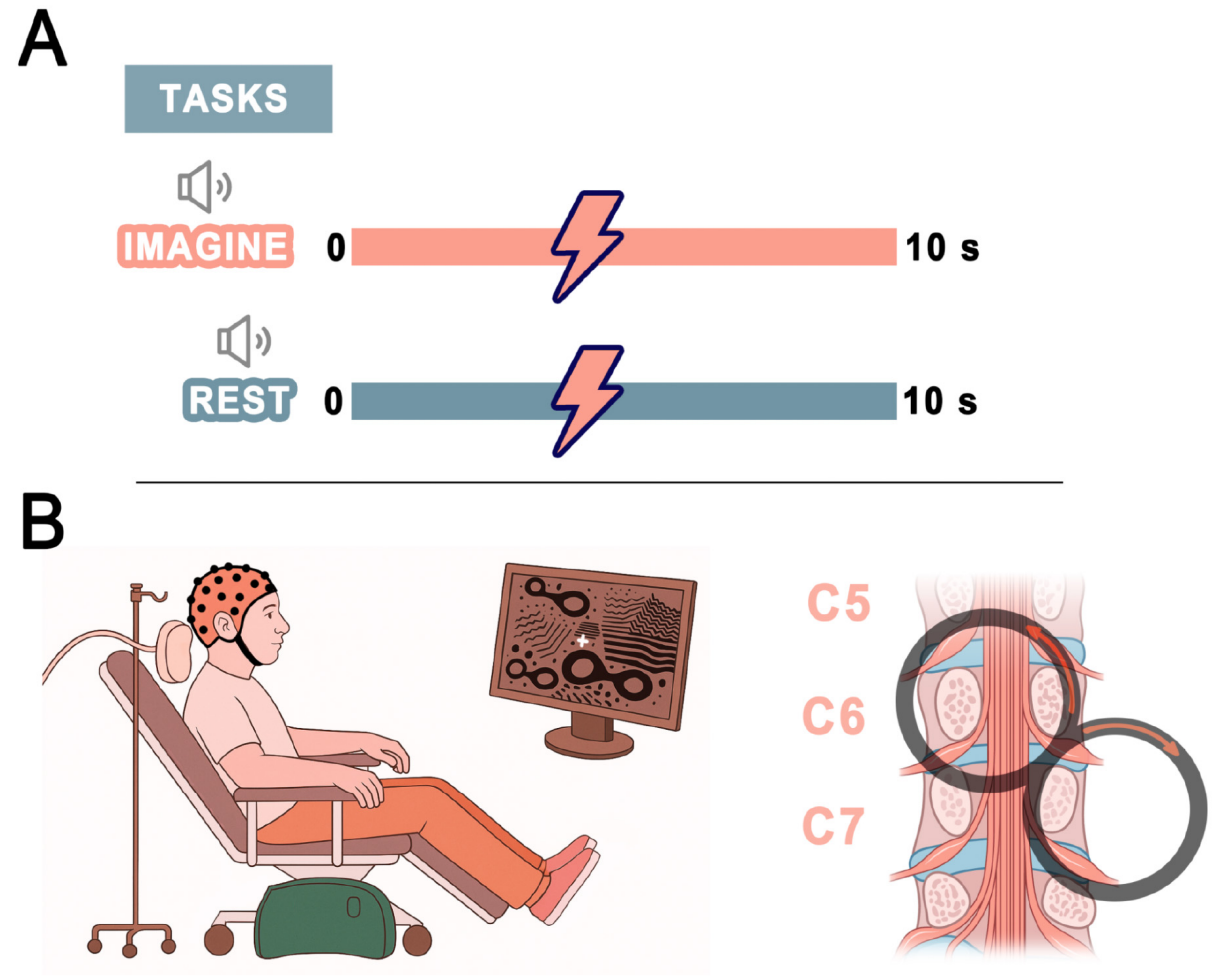
Experimental design. **(A)** Behavioral tasks. Each trial lasted 10 s. The magnetic pulse was delivered at a random time point 4–6 s after the auditory cue signaling the onset of the imagery task, ensuring that corticospinal excitability was probed when the mental image was expected to have reached maximal vividness. A total of 40 pulses were applied for each condition. **(B)** Schematic of spinal cord stimulation with a figure-of-eight coil positioned over the right side of the spinal cord on the right and, on the left, a participant setting during the experiment with their gaze fixed on a cross displayed on the screen.

Magnetic pulses were delivered at randomized time points between 4 and 6 s after the onset of the auditory cue signaling the start of the motor imagery task, while participants were actively engaged in imagery. This temporal jitter was intended to minimize anticipatory effects across trials and to ensure that stimulation probed excitability at a stage when motor imagery had reached a stable and vivid state. Each condition comprised 40 separate trials of 10 s duration, with one magnetic pulse delivered in each trial (Figure 1).

Participants were seated comfortably in a TMS chair with their eyes open and their gaze fixed on a cross displayed on a screen. An auditory cue instructed them either to begin motor imagery (MI) or to remain at rest. During MI trials, participants performed kinesthetic imagery of right-hand palm opening and closing. TMS pulses were delivered over the C6-C7 spinal level using a figure-of-eight coil (70 mm diameter; Nexstim, Helsinki, Finland), oriented at a 45° angle relative to the spinal cord. Spinal stimulation was delivered using biphasic pulses with a pulse duration of 280 μs, in accordance with the standard parameters of the Nexstim system. The C6-C7 vertebral level was identified using established anatomical landmarks. The stimulation hotspot was then localized by moving the coil slightly around the target area while monitoring MEP responses. The resting motor threshold (RMT), defined as the minimum stimulation intensity required to evoke MEPs >50 μV in 5 of 10 consecutive trials, was determined separately for each spinal stimulation site immediately before the corresponding stimulation condition. Stimulation intensity was set at 110% of RMT, and the order of conditions was randomized across participants.

#### EMG recording

Surface electromyographic (EMG) activity was recorded in four muscles: the right flexor digitorum superficialis (R_FDS), abductor digiti minimi (R_ADM), extensor digitorum communis (R_EDC), and the left flexor digitorum superficialis (L_FDS). EMG data were collected during all experimental conditions for further analysis. EMG was sampled at 5 kHz using the Nexstim EMG system.

### Experiment 2

In the second experiment, 20 volunteers (eight females, 12 males), aged 20–35 years (mean = 25.3, all right-handed), were recruited under the same inclusion and exclusion criteria as in Experiment 1. All participants gave informed consent prior to participation.

#### Experimental design and setup

Experiment 2 required participants to perform the same tasks as in experiment 1.

Here, single-pulse stimulation was applied over the left motor cortex (M1) and the right side of the spinal cord (R_TSMS). The experimental design was identical to experiment 1. Most participants were naïve to motor imagery paradigms and transspinal magnetic stimulation. Three participants from Experiment 1 also participated in Experiment 2. As in Experiment 1, participants were familiarized with the motor imagery task before the start of the session. The number of magnetic pulses/trials was 70 per condition. Coil position and orientation were controlled using the Nexstim navigation system (Nexstim, Helsinki, Finland) for both cortical and spinal-cord stimulation.

In addition to EMG recordings, electroencephalography (EEG) signals were recorded to investigate cortical responses to TSMS and TMS and their modulation by MI. TMS pulses were delivered using a Nexstim figure-of-eight coil, oriented at 45° with respect to the sagittal plane, single biphasic pulses with a pulse duration of 280 μs, consistent with the standard parameters of the Nexstim system (Nexstim, Helsinki, Finland). Neuronavigation was guided by the individual MRIs for each participant and the precentral area over the left motor cortex was targeted. A hot spot for FDS was first found, followed by RMT determination. For the experimental conditions, 120% of RMT intensity was used.

The right-side TSMS stimulation was organized the same way as in experiment 1. An adapted system was set up to ensure that the coil position remained stable over the spinal cord during stimulation.

#### EEG recording

EEG was recorded using a TMS-compatible NeurOne DC amplifier (Bittium Plc, Kuopio, Finland) with 62 passive Ag/AgCl flat electrodes positioned according to the standard 10-05 International System. The ground electrode was positioned on the forehead, and the reference electrode was placed at the FCz position. Electrode contact impedance was maintained below 5 kΩ. EEG data was sampled at 20 kHz and synchronized with TMS stimulus delivery. Participants wore earplugs during the procedure to minimize auditory artifacts from TMS clicks.

#### EMG data processing

EMG signals were epoched from −100 ms to +100 ms relative to the stimulation pulse, with the pre-stimulus period (−100 to 0 ms) serving as a baseline for correction. Pre-stimulus EMG activity was assessed by analyzing the first 100 ms of each epoch. Epochs with signal power exceeding the mean + 3 standard deviations for the pre-stimulus period were excluded (on average, 5% of epochs were excluded per participant per condition).

The peak-to-peak amplitude of MEPs was calculated for each individual epoch as the difference between the maximum and minimum values within a 40 ms analysis window starting 10 ms after the TMS pulse for M1 stimulation and 5 ms after the TMS pulse for spinal stimulation. Mean MEP amplitudes were then calculated for each participant and condition. MEPs latency was determined as the time of the strongest negative deflection in the EMG signal following the magnetic pulse for each individual epoch. Mean MEP latency was subsequently calculated for each participant and condition.

**Figure 2:**
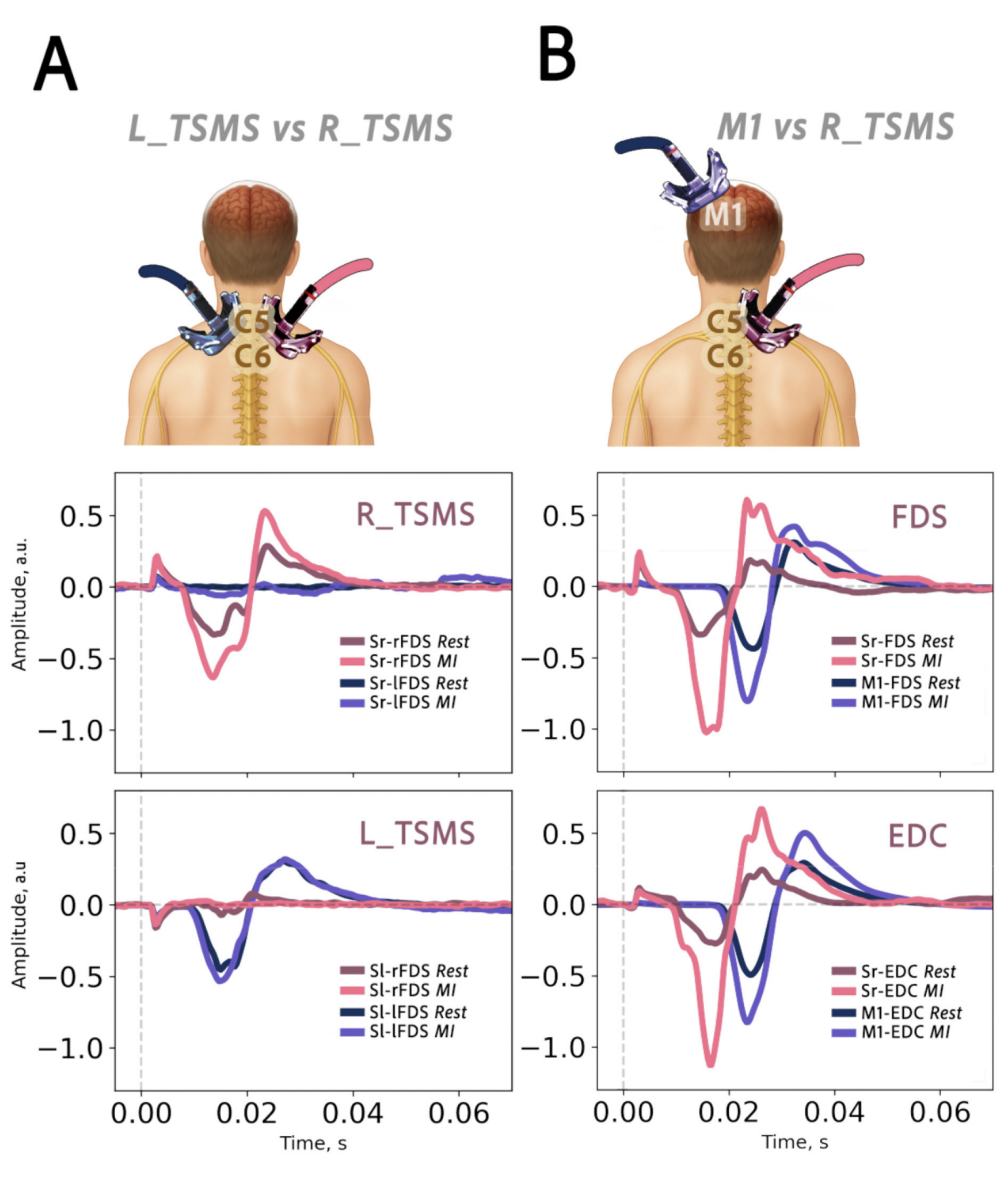
Motor-evoked potentials elicited by spinal (TSMS) and cortical stimulation (TMS). **(A)** Experiment 1: right and left side stimulation over the spinal cord during rest and imagery. **(B)** Experiment 2: M1 and right side spinal cord stimulation. R_TSMS, right side stimulation of the spinal cord; L_TSMS, left side stimulation of the spinal cord; M1, stimulation over the left motor cortex. FDS, *flexor digitorum superficialis*; EDC, *extensor digitorum communis*. Sr-FDS, stimulation right of right arm FDS; Sl-rFDS, stimulation left-right arm FDS; Sl-lFDS, stimulation of left side of the left arm FDS. For visualization purposes, MEP amplitudes were normalized by dividing each signal by its corresponding peak-to-peak amplitude during the rest condition. This normalization facilitated comparison of TSMS-evoked and M1-evoked motor potentials from different muscles on the same plot by better illustrating latency differences and MI effects while minimizing amplitude scale differences. The normalization procedure was applied separately for each stimulation type (TSMS and M1 TMS) and each muscle. This approach preserved within-stimulation differences between MI and rest while removing between-stimulation and between-muscle amplitude differences.

For visualization purposes and to account for differences in signal amplitude between muscles, MEPs were normalized to the peak amplitude obtained during the corresponding rest condition. This enabled the presentation of TSMS and TMS-evoked MEPs in combined figures (Figure 2).

**Figure 3:**
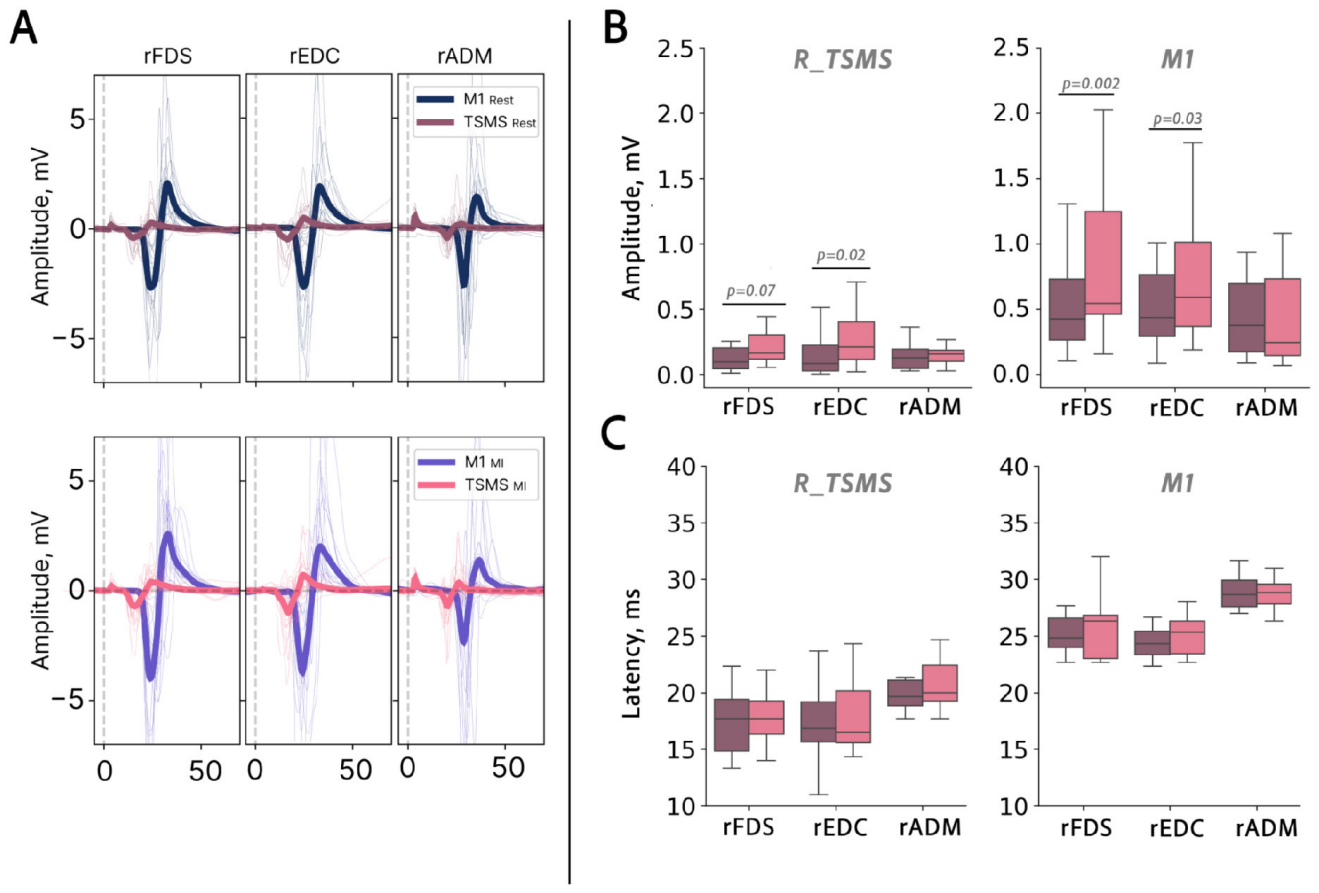
**(A)** TSMS and M1 stimulation evoked motor responses. Group-average and individual, non-normalized (raw) MEPs are shown for the MI and Rest conditions. Individual-subject MEPs are displayed as transparent lines, whereas group-averaged MEPs are shown as bold lines. **(B)** Peak-to-peak amplitudes of the motor-evoked potentials (MEPs) during rest and MI. Data are shown for different muscles and stimulation sites (R_TSMS vs. M1). **(C)** MEP latencies across conditions, stimulation sites, and muscles. Pink color shows MI and purple color Rest.

In experiment 2, to determine whether motor imagery differentially modulated M1-TMS and TSMS-evoked MEPs, we conducted separate 2-way repeated-measures ANOVAs for each muscle, with AREA (M1, TSMS) and CONDITION (Rest, MI) as within-subject factors. The stimulation AREA × CONDITION interaction was evaluated to test for differential modulation. *Post hoc* analyses were conducted using paired-samples *t*-tests. To control for multiple comparisons, *p*-values were adjusted using the Šidák correction (Figure 3).

To determine whether MEP latencies differed as a function of stimulation site, task condition, and recorded muscle, we performed a 3-way ANOVA with CONDITION (Rest, MI), MUSCLE, and STIMULATION AREA (M1, TSMS) as factors. The CONDITION × STIMULATION AREA interaction was evaluated to test whether motor imagery differentially affected MEP latencies elicited by M1 TMS and TSMS, whereas interactions involving MUSCLE were assessed to determine whether these effects differed between muscles (Figure 3).

#### EEG preprocessing pipeline

In the EEG analysis, EEG data were first downsampled from 20 to 10 kHz and epoched from −1,000 ms to 1,000 ms relative to the TMS pulse onset. The epochs with artifacts (approximately 2% of the epochs) were removed. Next, the signals within the interval −2 to 15 ms relative to TMS onset were replaced by interpolated values calculated using segment-wise linear interpolation (from the scipy.interpolate 1.10.1 package).

Independent Component Analysis (ICA) was performed in two stages. ICA-1 targeted artifacts directly related to TMS pulses, including residual stimulation noise and electrical noise decay around stimulation timing; components to exclude were identified visually and then excluded. On average, 7 components were deleted for each recording at this stage. A notch filter was only applied to a copy of the data before running the second ICA (ICA-2).

ICA-2 was performed to identify and remove components reflecting neurophysiological artifacts, including eye movements, blinks, muscle activity, and cardiac activity. Components marked for removal were identified by visual inspection and subsequently excluded. The corresponding excluded component weights were then applied to the copy of non-notch filtered data obtained after ICA-1.

**Figure 4:**
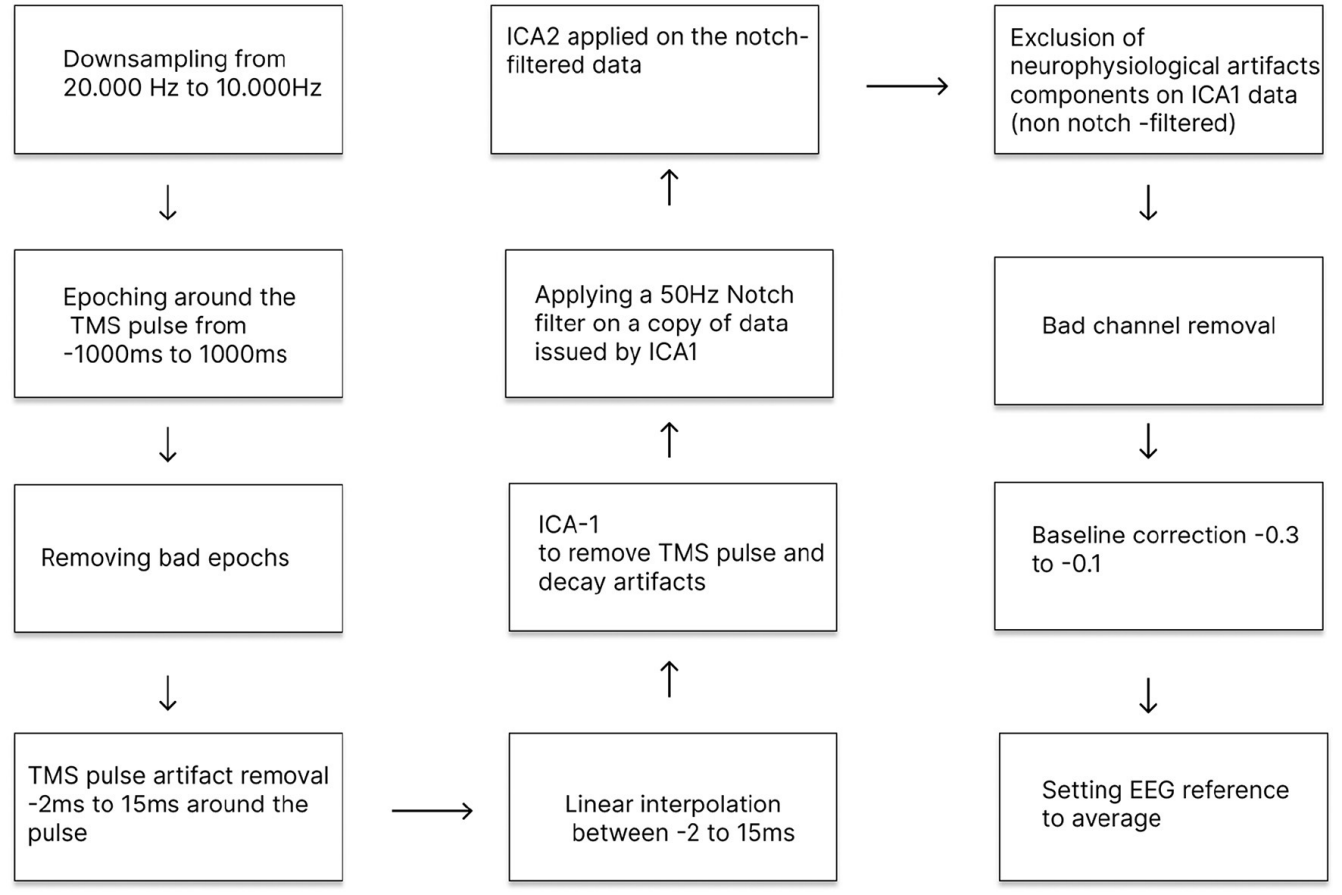
EEG preprocessing steps, including removal of TMS-related and physiological artifacts and subsequent analysis of TMS-evoked potentials.

Next, after the two rounds of ICA, a notch filter at 50 Hz was applied to suppress power line noise, followed by band-pass filtering (0.1–45 Hz). Noisy channels were marked and removed, then interpolated, followed by a re-referencing to the average reference (common average reference), and corrected to the baseline measured for the interval −0.3 to −0.1 s relative to the trial start. ICA and filtering were implemented using the MNE Python library (3.11.3). Figure 4 shows the graphic representation of the signal preprocessing pipeline.

#### TMS and TSMS cortical evoked potentials analysis

In total, we had *N* = 17 for R_TSMS MI, *N* = 19 for R_TSMS_Rest, *N* = 19 for M1_MI, and *N* = 18 for MI_Rest files to analyze.

To assess the spatiotemporal dynamics of stimulation-evoked activity, epochs were averaged for each participant across both motor imagery and rest conditions, then grand averages were computed across participants, and topographical maps were generated for different components from 15 to 300 ms post-TMS pulse. Time points for the topographical maps were selected based on visual inspection of TEPs and TSEPs peaks with reference to the established temporal structure of TMS-evoked potentials reported in the literature (30, 60, 100, 180, 200, 280, and 300 ms).

To identify significant differences in TEPs and TSEPs between MI and rest conditions, a spatiotemporal cluster-based permutation test was applied to the evoked cortical signals within the 15–350 ms interval (the interpolated time range from 0 to 15ms was excluded from statistical analysis). Statistical comparisons were conducted using a two-sided cluster-based permutation approach with 2,000 permutations, using a threshold of *t* = 2.5. Clusters with *p*-values ≤ 0.05 were considered statistically significant. For visualization, mean F-statistic maps were plotted alongside averaged condition-wise waveforms over significant channels.

All data processing and statistical analyses were conducted in Python (version 3.10). Signal preprocessing was performed using MNE-Python (version 1.9.0). Cluster-based permutation statistics were computed using the mne.stats.spatio_temporal_cluster_test function (Maris and Oostenveld, 2007). Repeated-measures ANOVA was implemented using the Pingouin library (version 0.5.5; Vallat, 2018).

## Results

### Analysis of motor evoked potentials

EMG analysis demonstrated that TSMS selectively activated side-specific motoneuron pools, eliciting unilateral hand contractions. Figure 2A shows the results of experiment 1, where R_TSMS (coil positioned over the right side of the spine) and L_TSMS (coil positioned over the left side of the spine) stimulation evoked muscle activation in the ipsilateral hand. MEP amplitudes exhibited similar latencies and morphology across stimulation sites for all recorded muscles. However, right-hand movement imagery increased right-hand MEP amplitudes in R_TSMS, while amplitudes of MEPs evoked by L_TSMS remained unchanged between conditions.

In the second experiment, we compared motor responses between TSMS and M1 stimulation. Figure 2B demonstrates that both stimulation conditions exhibited increased MEP peak-to-peak amplitudes during MI; moreover, a difference in latency was found, where MEPs evoked by TSMS occurred earlier than MEPs evoked by M1 stimulation.

To determine whether MI differentially modulated MEP amplitudes across stimulation sites and muscles, separate two-way repeated-measures ANOVAs were conducted for each muscle, with CONDITION (rest, MI) and AREA (M1, R_TSMS) as within-subject factors (Figure 3).

For the right flexor digitorum superficialis (R_FDS), both main effects were significant: MI increased MEP amplitudes relative to rest [*F*_(1, 15)_ = 6.94, *p* = 0.019, η^2^g = 0.076], and M1 stimulation evoked larger MEPs than TSMS overall [*F*_(1, 15)_ = 40.65, *p* < 0.001, η^2^g = 0.446]. The AREA × CONDITION interaction did not reach significance [*F*_(1, 15)_ = 3.21, *p* = 0.093, η^2^g = 0.034], indicating that the facilitatory effect of MI on MEPs amplitude was present regardless of stimulation site. For the right abductor digiti minimi (R_ADM), no significant main effect of condition was observed [*F*_(1, 13)_ = 0.016, *p* = 0.900, η^2^g < 0.001], while responses differed significantly between stimulation sites [*F*_(1, 13)_ = 5.80, *p* = 0.032, η^2^g = 0.172]; the interaction was non-significant [*F*_(1, 13)_ = 0.73, *p* = 0.409]. A similar pattern emerged for the right extensor digitorum communis (R_EDC), where only the main effect of stimulation site reached significance [*F*_(1, 15)_ = 12.93, *p* = 0.003, η^2^g = 0.219], with neither the main effect of condition [*F*_(1, 15)_ = 3.06, *p* = 0.101] nor the interaction [*F*_(1, 15)_ = 0.46, *p* = 0.510] reaching significance (Figure 3A).

The *post hoc t*-tests showed a significant difference between MI and rest for M1-TMS for R_FDS (*t* = 3.61, *p* = 0.002) and R_EDC (*t* = 2.34, *p* = 0.03). R_EDC also showed significant increase for TSMS (*t* = 2.55, *p* = 0.02). No significant differences were observed in TSMS stimulation for R_FDS (*t* = 1.88, *p* = 0.07), R_ADM (*t* = 1.48, *p* = 0.16; Figure 3A).

On the other hand, MEPs latencies showed a significant effect of stimulation site (TSMS vs. M1; F = 24.74, *p* < 0.000001, η^2^ = 0.107; Figure 3B), and a significant effect of muscle [*F*_(2, 206)_ = 11.08, *p* < 0.001, η^2^ = 0.097], suggesting varying latencies across stimulation sites and muscles. The condition (MI vs. rest) had no significant effects on MEPs latency (*p* = 0.231) in our study.

No normalization was applied for statistical analyses, which was based on raw MEP values using paired within-subject comparisons of MI-related changes.

### EEG analysis

**Figure 5:**
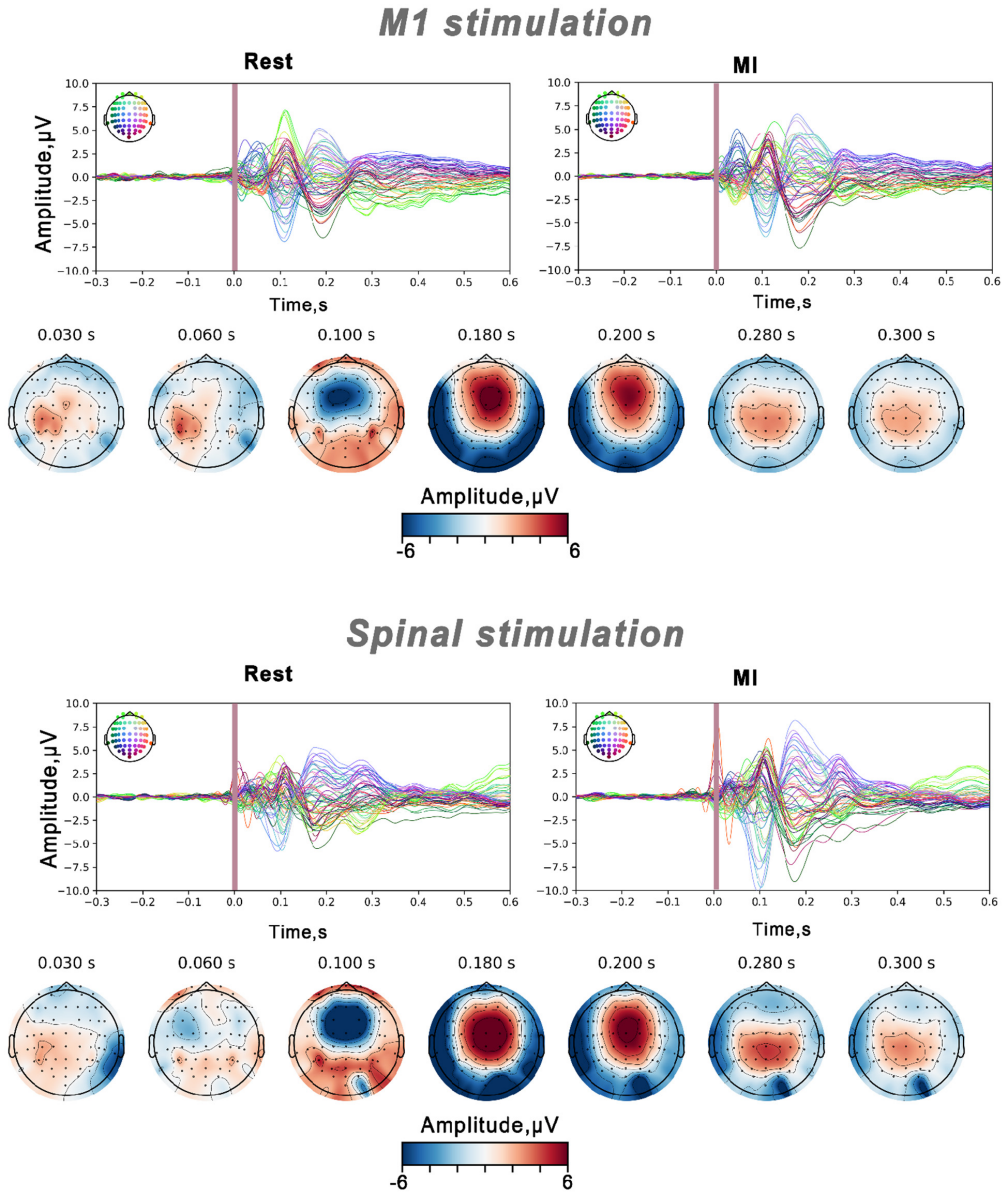
Groupe average TMS-evoked potentials (TEPs) and TSMS-evoked potentials (TSEPs) across all channels during the rest and right-hand MI conditions. The TMS artifact interval from −2 to +15 ms is indicated by purple shading. Scalp distribution of specific peaks is shown as topographic maps during MI conditions.

M1 stimulation-evoked TEPs waveforms matched the classical TEPs reported in the literature (Beck et al., 2024), exhibiting P30 and P60 peaks followed by N100 and P180 components. Early peaks demonstrated left-sided scalp localization, while later (180–300 ms after stimulus onset) components showed central-parietal distribution (see Figure 5). On the other hand, the TSMS-evoked cortical potentials also exhibited an early P30 and, in contrast to M1, a negative deflection at 60 ms, with scalp distribution contralateral to the stimulated spinal side. N100 and later components were localized to central-parietal channels.

**Figure 6:**
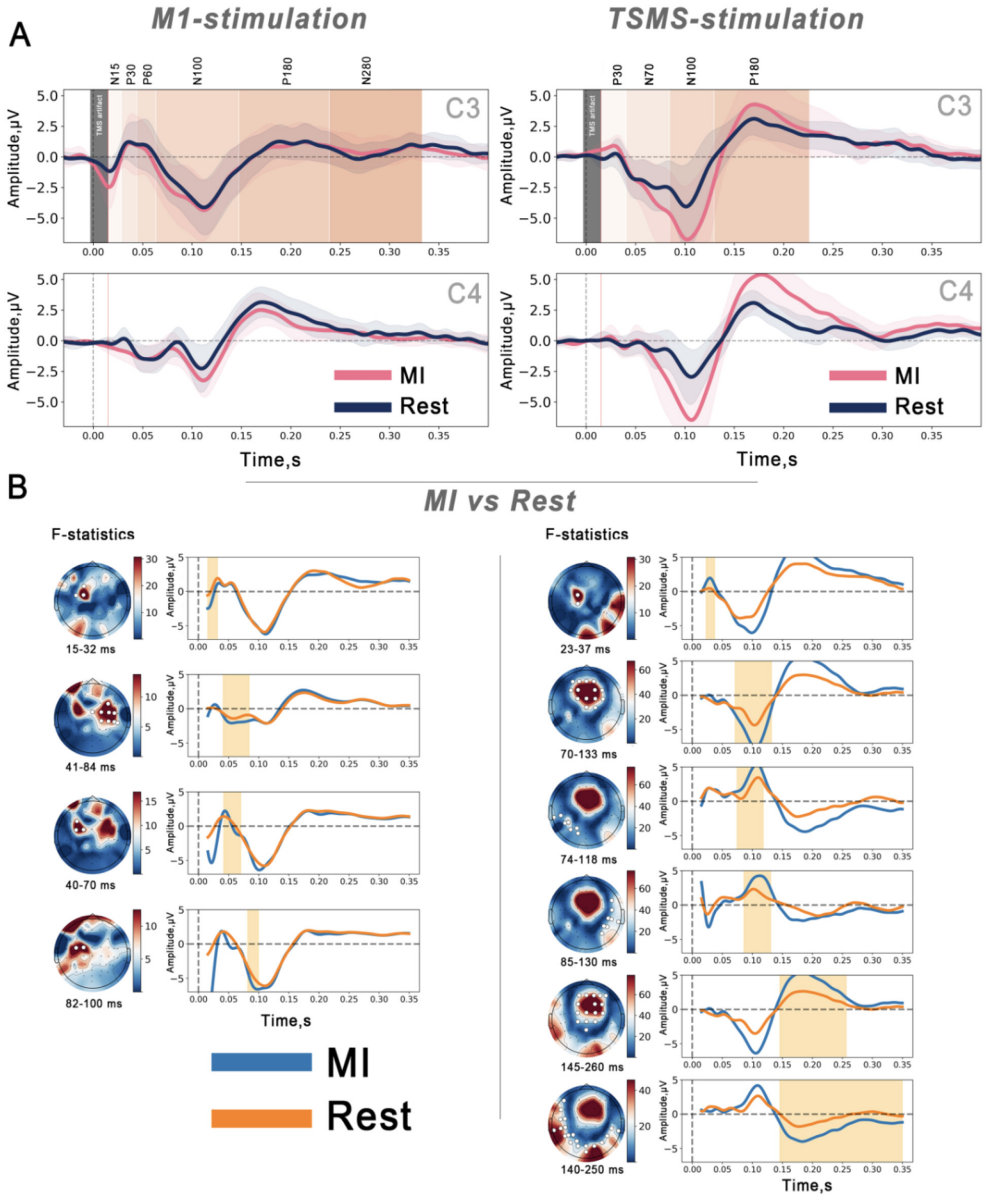
**(A)** TMS-evoked potentials (TEPs) and TSMS-evoked potentials (TSEPs) over channels C3 and C4 during MI and rest at a group level. **(B)** Cluster-based permutation test showing significant clusters corresponding to TEPs and TSEPs peaks during MI compared to rest at a group level.

The cluster-based permutation test revealed multiple spatio-temporal clusters showing MI effects for both cortical responses, as seen in Figure 6. For TEPs, significant clusters included an early cluster over the stimulated left motor cortex at 15–20 ms post-stimulus (N15), two clusters (ipsilateral and contralateral to the stimulation side) corresponding to the P30 component, and a late cluster at ~100 ms post-stimulus corresponding to the N100 peak. All clusters indicated MI-induced increases in TEPs component amplitudes compared to rest.

For TSEPs, MI also increased component amplitudes. The permutation test identified an early cluster contralateral to the stimulated spinal side corresponding to the P30 component (23–37 ms) around the sensorimotor area and later significant clusters (70–144 ms) and (145–280 ms) over the centroparietal and frontal areas.

## Discussion

In this study, we used TMS applied to M1 and TSMS within the same participants to directly characterize MI's modulation of both cortical and spinal excitability. First, we report that TSMS-evoked MEPs exhibited significantly shorter latencies than M1-evoked MEPs (*p* < 1e-6), providing direct evidence that TSMS can activate spinal circuits independently of cortical signaling. Second, right-hand kinesthetic MI selectively enhanced TSMS-evoked MEPs the ipsilateral stimulated side but not contralaterally, demonstrating that MI modulates spinal cord excitability in a muscle effector-specific manner. Third, TSMS generated ascending cortical evoked potentials (TSEPs) that showed significantly higher activation during MI, revealing that motor imagery also enhances the cortical processing of afferent spinal volleys. Together, these findings provide the first direct magnetic evidence of a bidirectional MI-modulated brain-spinal cord loop, with kinesthetic imagery facilitating both descending motor activation at the spinal level and ascending sensorimotor processing at the cortical level simultaneously.

### TSMS MEPs' amplitude increases during MI

With TSMS, we reliably elicited MEPs using a figure-of-eight coil positioned at the right side of the spine between the C6–C7 vertebrae and oriented at ~45°. One previous study demonstrated that coil position during TSMS affected the laterality and focality of muscle responses (Ren et al., 2024). We confirmed this spatial specificity by showing that by positioning the coil on the right or left side of the spine MEPs could be evoked exclusively in the ipsilateral upper limb (Experiment 1).

Additionally, MI of the right hand being closed and opened selectively enhanced the R_TSMS-induced MEPs but not the L_TSMS-induced MEPs. This task facilitation can be compared to the literature on the effects of MI on TMS-evoked responses demonstrating that these effects are muscle- or effector-specific (Fadiga et al., 1998; Kaneko et al., 2014; Wright et al., 2014). Here we extend this by showing that such task specificity during MI is extended to the spinal cord. In Experiment 2, we also found that MI increased muscle responses evoked by M1 TMS, which is consistent with the previously established facilitatory action of MI on corticospinal excitability (Kasai et al., 1997; Facchini et al., 2002; Fadiga et al., 1998). Notably, we observed that TMS evoked stronger MEPs amplitudes compared to the responses to TSMS. This could be explained by methodological factors such as the assurance that TMS-stimulated M1 was guided by subject-specific MRI neuronavigation and delivered at 120% RMT, while the spinal stimulation was conducted at 110% RMT without MRI-anatomical guidance. Additionally, the relative weakness of the TSMS-evoked responses does not speak against this approach, particularly if TSMS activates different motoneuronal pools compared to TMS. Further investigation of optimal stimulation intensity will be needed for TMS, TSMS, and their combinations.

In addition, regarding muscle responses, all muscles showed a numerical increase in their MEPs amplitude during MI; however, the ADM muscle didn't reach statistical significance in both cortical and spinal stimulation conditions, which might be explained by weak task relevance relative to the FDS and EDC on hand opening and closing, which confirms the muscle effector effect literature (Fadiga et al., 1998; Hara et al., 2010; Stinear and Byblow, 2003).

Furthermore, TSMS-evoked MEPs had significantly shorter latencies than TMS-evoked MEPs (area effect: *p* < 1e-6); this result is expected due to the shorter conduction path for the spinal reflexes evoked by the stimulation of dorsal roots compared to the corticospinal tracts. Differences in muscle-specific motoneuron pool anatomy also might have contributed to latency variability (muscle effect: *p* < 0.001). However, we report no task-dependent significance in latency, as MEP latencies remained unchanged during MI compared to rest (*p* = 0.231).

Whether MI significantly modulates spinal circuitry has long been debated, with classical reflex measures showing inconsistent results, which was likely due to the limitations of monosynaptic reflexes and presynaptic gating mechanisms. Our study demonstrated that TSMS-evoked MEPs had significantly shorter latencies than M1-evoked MEPs, directly establishing the spinal origin of our evoked responses and confirming that imagery modulates the spinal cord excitability in an effector-specific manner. This side- and muscle-specificity confirms the well-established effector specificity of MI-related corticospinal facilitation (Fadiga et al., 1998; Kaneko et al., 2014; Wright et al., 2014). In general, our findings indicate that the descending signal produced during kinesthetic motor imagery is not limited to cortical circuits; rather, it extends along the corticospinal tract to actively influence motoneuronal pool excitability in the spinal cord in a spatially organized and task-relevant manner.

### Cortical responses to spinal and motor cortex stimulation

#### TMS-EEG data preprocessing pipeline

EEG recorded with concurrent TMS or TSMS is highly susceptible to artifacts. Beyond traditional physiological artifacts including muscle activity and eye movements, the magnetic pulse itself induces substantial distortions in the EEG signal, followed by post-pulse decay artifacts. To ensure valid TEPs and TSEPs measurements, we implemented a multi-stage preprocessing pipeline with carefully ordered steps: interpolation around the pulse artifact and two rounds of ICA with component exclusion guided by topography and trial-to-trial variability.

As several prior studies have shown that the preprocessing approach and step order influence early TEP waveforms (Hernandez-Pavon et al., 2022, 2023; Ilmoniemi and Kičić, 2010; Rogasch et al., 2017; Brancaccio et al., 2024; Bertazzoli et al., 2021). Indeed, when identical data were processed through four different pipelines, components before 100 ms showed divergence in amplitude and waveform, while late components demonstrated high inter-pipeline correlation (Bertazzoli et al., 2021). Recent work by (Brancaccio et al. 2024) comparing ARTIST, TESA, and SOUND/SSP-SIR using synthetic ground-truth data revealed substantial differences in spatial-temporal precision and inter-trial variability. Therefore, we implemented our pipeline conservatively to preserve early neural activity while minimizing artifact contamination.

#### TMS and TSMS evoked potentials

The topography of the TMS-evoked potentials (TEPs) in our study was consistent with previous reports. The TEPs displayed early components (P30 and P60) localized over the stimulated motor cortex, as well as later components (N100 and P180) with a broader distribution (Beck et al., 2024; Brancaccio et al., 2024) during rest and MI conditions. We observed that MI increased the amplitude of the early TEP components within the 15–32 ms interval after the magnetic pulse (see Figure 6). This aligns with the prior studies reporting that MI enhances cortical excitability (Fadiga et al., 1998; Pfurtscheller and Neuper, 1997; Ahn and Fröhlich, 2021) as the P30 component is considered to reflect early motor cortex activation. The later TEP components displayed bilateral activations, suggesting the involvement of the ipsilateral sensorimotor networks during a unilateral motor task and reflecting a broader cortical engagement that extends beyond the contralateral hemisphere (Beck et al., 2024; Ahn and Fröhlich, 2021; Syrov et al., 2024).

Furthermore, we report that TSMS delivered at the cervical level elicits cortical responses (TSEPs) that resemble canonical TEP waveforms evoked by M1 TMS. Early TSEP peaks in the 20–40 ms range may correspond to fast somatosensory afferents and local sensorimotor processing (Beck et al., 2024; Ahn and Fröhlich, 2021; Syrov et al., 2024; Fecchio et al., 2017; Zhang et al., 2024). The presence of the P30 component over this area in TSEPs suggests the activation of sensorimotor circuits even when the stimulation pulse originates at the spinal level.

However, a difference emerged in the 60–70 ms time window, where TSEPs showed a negative deflection (N60), whereas M1-evoked TEPs typically display a positive peak at this latency. This polarity inversion could reflect distinct neural processes and pathways engaged by the spinal vs. cortical stimulation; in one M1 TMS study, the P60 component was hypothesized to reflect afferent input from the stimulated M1 region to the primary somatosensory cortex (S1), likely arising from muscle twitches induced by the TMS pulse and subsequently processed by S1. Another source localization study has localized the P60 peak to the left superior parietal lobule, associated with S1 processing (Beck et al., 2024; Fecchio et al., 2017; Zhang et al., 2024).

To our knowledge, no study has investigated the temporal characteristics of the waveform components evoked by magnetic spinal stimulation here. Although the TEPs waveforms are similar between the two stimulation areas, the topographic maps show a slight positive deflection at 60 ms on the cortex during spinal stimulation, which might be explained by different latencies in processing and/or differences in the network activation. Further source localization analysis will be needed to determine whether early TSEP components represent purely sensory evoked potentials or also reflect corticospinal network activity and to identify the neural sources activated during different time windows under TSMS conditions.

### Motor imagery enhances cortical responses to magnetic spinal stimulation

Another key finding in our study is that MI modulated the cortical responses to the efferent magnetic spinal stimulation by significantly increasing TSEPs amplitudes, particularly at the P30 component (Beck et al., 2024; Steele et al., 2022; Gruenwald et al., 2025), indicating a possible cognitive priming of the motor cortex enhances early cortical processing of ascending spinal volleys.

TSEPs also exhibit higher activations in C3 and C4 channels (see Figure 6A) compared to M1; this process might be attributed to the TSMS approach generating afferent volleys that ascend to the cortex, activating not only the sensory tracts but also motor neurons and other spinal interneuronal circuits; however a formal statistical comparison between TEPs and TSEPs amplitudes and a source localization analysis would be needed to fully characterize this difference between the evoked potentials produced by the two stimulation sites.

### Novelty

Focal TSMS presents a promising approach for side-specific rehabilitation targeting, potentially enhancing therapeutic outcomes. This study offers several novel insights into the neural mechanisms underlying TSMS alone and combined with MI. While the effects of motor imagery on cortical activity are well-documented, to our knowledge this is the first study to investigate the spatiotemporal neural correlates of magnetic spinal stimulation during MI using EEG. We meticulously characterized the EEG correlates of TSMS and identified distinct TEP components, revealing similarities and differences in waveform components to those observed in cortical stimulation after rigorous preprocessing. Moreover, in a within-subject paradigm, we demonstrate that TSMS responses exhibit different latencies in motor pathways (as measured by EMG and possibly distinct spatial cortical activations) compared to M1 stimulation and a reciprocal connection between the brain and spinal cord during MI.

### Limitations and future directions

Further investigation into the neural mechanisms of TSMS is required; employing source localization techniques and advanced EEG analyses will be crucial to accurately map TSMS-evoked potentials on the cortex and understand their underlying neural substrates. Indeed, while our findings suggest that lateralized TSMS coil positioning can evoke ipsilateral responses, we cannot conclusively assert that this specific coil orientation and positioning achieve the most precise root-specific stimulation without additional position mapping and electrical field modeling studies. Similarly, optimal stimulation intensity needs to be investigated to minimize shoulder or movement artifacts as well as discomfort for participants while maximizing stimulation output.

It is also important to note that TEP waveforms can be influenced by peripheral sensory inputs and artifacts. Therefore, meticulous preprocessing and control conditions are essential to isolate genuine cortical responses. In our study, we applied rigorous EEG preprocessing to minimize such confounds; however, further refinement of TMS-EEG pipelines remains an active area of research (Hernandez-Pavon et al., 2022, 2023; Ilmoniemi and Kičić, 2010; Rogasch et al., 2017; Brancaccio et al., 2024; Bertazzoli et al., 2021).

Another limitation of this study is that there were no formal questionnaires (e.g., KVIQ or MIQ-RS) were used to quantify imagery vividness, future studies should consider adding MI questionnaires or online monitoring of MI via EEG to verify task compliance throughout the entire stimulation interval.

Combining TSMS with MI could collaboratively enhance neuroplasticity by synchronizing cortical and spinal activations, potentially improving recovery in patients with SCI or after a stroke, and the optimization of simulation parameters for magnetic stimulation of the spinal cord with TMS, including coil orientation and stimulation intensity, requires further investigations to improve the precision and efficacy of TSMS protocols within the clinical population.

## Conclusion

Spinal cord stimulation techniques have demonstrated promising modulatory effects for patients with SCI and stroke, contributing to a decrease in spasticity, alleviation of pain, and improvements in motor functions (Hordacre et al., 2020; Capozio et al., 2025; Gorgey et al., 2022; Kathe et al., 2022). TSMS emerges as a promising method not only for probing central nervous system excitability but also for serving rehabilitative purposes. The current study provides insights for future rehabilitative protocols exploring the combination of motor imagery with TSMS, leveraging both the top-down priming of the motor cortex by MI and the bottom-up effects of spinal stimulation to the cortex.

## Data Availability

The raw data supporting the conclusions of this article will be made available by the authors, without undue reservation.
